# Supplementary material for: The epidemiology and management of chronic osteomyelitis in pediatrics – A systematic review
Source: PLoS One. 2025 Dec 2;20(12):e0337516. doi: 10.1371/journal.pone.0337516 (PMC12671803; doi:10.1371/journal.pone.0337516)
Supplement: S1 File — This is the strategy for searching the literature. (PDF) [file pone.0337516.s001.pdf]

Data Exports, Reports, and Stats

Number of results returned: 42
Total number of records queried: 42

Selected instruments (all records)

**Record ID** record_id	**Year** yr_v2	**First author** author_v2	**Country of author** country_v2	**Country of origin of patients** country_pts	**Were any managed with antibiotics alone?** antibiotics_alone	**Were any only on PO antibiotics?** po_only	**Were any not given antibiotics?** no_antibiotics	**Which is this?** type_study	**Definition used for chronic osteomyelitis** defn	**Consecutive cases** consecutive	**Are any cases o chronic osteom related surgica placed foreign body?** fb
02	2024	Al-alawi	Oman					epi study from wealthy country (1)	"persistence or recurrence of attributable symptoms and signs associated with a sequestrum, involucrum or osteosclerosis on a plain radiograph, requiring antibiotics for at least 12 weeks."	yes (1)	No (0)
05	2023	Mulualem	Ethiopia					epi study from poor country (2)	6 weeks of clinical signs and evidence of one or more of the following radiological findings: sequestrum, involucrum, soft tissue swelling that obliterates the fat planes, periosteal reaction, lytic destructions, and cloaca. Other findings that were used to diagnose a case as COM in our study were either extensive sclerosis or Brodie's abscess	yes (1)	No (0)
06	2019	Edson	Uganda						none in Methods but intro says "Chronic osteomyelitis (COM) is a type of osteomyelitis that is relapsing and persistent characterized by low grade inflammation, presence of sequestrum, involcrum, brodies abscess and fistulous tracts"	yes (1)	No (0)
07	2015	Stevenson - Beckles (2 papers)	Malawi		Stevenson states some cases were but in Table 3 the 7 patients had 0.9 ORs each so maybe only one - Beckles says all had ORnly wsesesIt	Peri-operative intravenous antibiotics were administered to most pa tients, with longer courses being prescribed on clinical grounds.	no	epi study from poor country (2)	Beit CURE Classification	yes (1)	No (0)

09	2013	Ponio	Phillipines		8%	not stated	1 ?CRMO	epi study from poor country (2)	Chronic osteomyelitis was used to describe an infection when symptoms have been present for more than three weeks at the time of presentation with radiologic findings of sequestration, bone destruction and cloaca formation.1	no (2)	No (0)
10	2011	Mantero	Kenya		no	no	n0	epi study from poor country (2)	Solagberu stage III [21], Cierny-Mader stage I-IV [20], symptoms of osteomyelitis persisting for at least 6 months, evidence of fistula tract, and radiological evidence of bone sequestration. In all these cases, there was indication for surgical treatment, medical treatment alone not being resolutive. According to Solagberu classification, all our patients were stage III. According to Cierny-Mader, 5 patients were group I (5,2%), 4 group II (4,2%), 76 group III (79,2%), and 11 group IV (11,5%). Exclusion criteria were symptoms of osteomyelitis for less than 6 months, no evidence of fistula, or no radiological signs of bone sequestration.	not stated (3)	No (0)
11	2023	Disch	US		yes - 82% were	not known	not stated	epi study from wealthy country (1)	ICD10 codes		
12	2022	Lazzeri	Italy		no	no	no	treatment trial (3)	NR	yes (1)	No (0)
13	2021	Kojima	Brazil		no	no	no	treatment trial (3)	drainage from fistula for at least 2 months	not stated (3)	Yes (1)
14	2018	Munshi	Fiji		not stated	not stated	not stated	epi study from poor country (2)	none	yes (1)	No (0)

15	2021	McNeil	US		yes	no	no	epi study from wealthy country (1)	(1) symptoms suggestive of osteomyelitis (eg, pain, swelling, warmth, erythema, drainage, loss of function, etc.) lasted ≥28 days on presentation or (2) there was a clearly documented history of acute osteomyelitis in a patient who received at least 4 weeks of effective antimicrobial therapy along with (a) new or worsening drainage, swelling, erythema, pain or loss of function; (b) radiographic evidence of sequestrum or permeative lucencies; or (c) readmission for the management of osteomyelitis.	yes (1)	Yes (1)
16	2019	Andreacchio	Italy		no	no	no	treatment trial (3)	none	yes (1)	No (0)
17	2004	Yeargan	US	6 from US; 33 from Pacific Islands; 1 from Korea	no	no	no	epi study from poor country (2)	duration > 6 months	yes (1)	
18	2014	Wirbel	Afgahnistan/ Angola (surgery in Germany)	Afghanistan/ Angola	no	no	no	epi study from poor country (2)	duration > 6 months		No (0)
19	2010	Bar-On	Israel		no	no	no	treatment trial (3)	none	not stated (3)	Yes (1)
21	2005	Matzkin	Children of Pacific Island ethnicity cared for in US	Shriners hospital - only counted those from Pacific Islands	yes	no	no	epi study from poor country (2)	none	yes (1)	No (0)

23	2000	Reinehr	Germany		yes	no	no	epi study from wealthy country (1)	(history of slight localized pain and/or swelling for at least 2 weeks) Note: some might be subacute	yes (1)	No (0)
24	2002	Paley	US		no	no	no	treatment trial (3)	none	not stated (3)	Yes (1)
25	2018	Rousset	France		no	no	no	treatment trial (3)	based on imaging but criteria not provided - all had infected non-union	yes (1)	Yes (1)
26	2001	Rasool	South Africa		no	NR	NR	epi study from poor country (2)	none	yes (1)	
28	1994	Lauschke	Namibia		no			epi study from poor country (2)		yes (1)	

30	1995	Bassey	Nigeria		no	no	no	epi study from poor country (2)	. Confirmation of diagnosis was based on X-ray featives such as the presence of sequestra and new bone formation, Brodie's abscesses and bone sclerosis at the site of osteomyelitis.	yes (1)	No (0)
31	2015	Costa	Portugal		yes	maybe	no	epi study from wealthy country (1)		no (2)	No (0)
32	2024	Bhattacharyya	India		no	no	no	epi study from poor country (2)	all cases treated with calcium sulfate beads (which can have gentamicin, tobramycin or vancomycin and do not require removal)	not stated (3)	No (0)
33	2018	Omoke	Nigeria		not stated	not stated	not stated		infection last >6 weeks with radiological evidence of sequestrum, sclerosis, and osteomyelitis associated with foreign bodies	yes (1)	
34	2011	Ulug	Turkey					epi study from wealthy country (1)	a bone infection that was worse or had not improved clinically or microbiologically after ≥ 10 days of evolution, independent of the presence or absence of surgical and / or antimicrobial therapy but only included those with sinus tracts		
35	2025	Alihinai	US		yes - 3/73	yes - 3/73	no	epi study from wealthy country (1)	Cases were classified as hardware-associated osteoarticular infection (HOI) if clinical and radiographic evi dence supported an OAI associated with hardware, regardless of the route of infection or duration of preceding symptoms.I included only hardware associated cases aS combined sub-acute with chronic	yes (1)	Yes (1)
36	2009	Akakpo-Numado	Togo		NR	NR	NR	epi study from poor country (2)	itwascalledchronic inpresenceof sequestrum and/or fistulawithchronic suppuration	not stated (3)	
37	2005	Beslikas	Greece		no	no	no	treatment trial (3)			No (0)
38	2021	Ellur	India		no	no	no	epi study from poor country (2)	hematogenous only	yes (1)	No (0)

39	2002	Bahebeck	Cameroon		no	no	no	epi study from poor country (2)	none but had to need surgery	yes (1)	No (0)
40	2006	Unal	Turkey		no	no	no	epi study from wealthy country (1)	none - all but 3 had draining sinuses	not stated (3)	No (0)
41	2018	Akyuz	Turkey		no	no	no		all had strenocutaneous fistulas but no definition provided	yes (1)	No (0)
42	2024	Peshin	India		no	no	no	epi study from poor country (2)	pus discharge from an extremity persisting for more than six weeks, along with radiological features indicative of chronic osteomyelitis.	no (2)	No (0)
43	2015	Shukrimi	Malaysia		yes - one of 3	no	no	epi study from poor country (2)		not stated (3)	No (0)
44	1997	Vogely	Netherlands		no	no	no	epi study from wealthy country (1)	table differentiates acute from chronic but criteria not definitive	not stated (3)	No (0)

45	2023	Shi	China		no	no	no	epi study from wealthy country (1)	chronic osteomyelitis of long bones, which was confirmed by clinical features and imaging (plain radiographs, CT, and MRI	yes (1)	
46	1991	Onuba	Zimbabwe					epi study from poor country (2)		not stated (3)	
47	2010	Zeng	China		no	no	no		based on the clinical findings and histopathology.	not stated (3)	
48	2008	Dieckmann	Germany		yes	no	no	epi study from wealthy country (1)	histopathology differentiated chronic from acute	not stated (3)	No (0)
49	1989	Saighi	Algeria		no	not stated	no	epi study from poor country (2)	none but all had fistulas		No (0)
54	1991	Tudisco	Italy		10	no	no	epi study from wealthy country (1)	Tachdjian	no (2)	No (0)
