# Supplementary material for: The epidemiology and management of chronic osteomyelitis in pediatrics – A systematic review
Source: PLoS One. 2025 Dec 2;20(12):e0337516. doi: 10.1371/journal.pone.0337516 (PMC12671803; doi:10.1371/journal.pone.0337516)
Supplement: S3 File — This is the data as it was entered into REDCap. (DOCX) [file pone.0337516.s003.docx]

**Supplement – Search methodology**

**Ovid MEDLINE(R) ALL <1946 to February 12, 2025>**

Date searched: Feb 13, 2025

Results: 694

1 ((recurrent or chronic or persistent) adj3 osteomyelitis).mp. 5120

2 exp Child/ or exp infant/ or adolescent/ or exp pediatrics/ or minors/ or (pediatric* or paediatric* or child* or newborn* or congenital* or infan* or baby or babies or neonat* or pre-term or preterm* or premature birth* or NICU or preschool* or pre-school* or kindergarten* or kindergarden* or "elementary school*" or "nursery school*" or ("day care*" not adult*) or schoolchild* or toddler* or boy or boys or girl* or "middle school*" or pubescen* or juvenile* or teen* or youth or youths or "high school*" or "junior high" or adolesc* or young-adult* or pre-pubesc* or prepubesc*).mp. 5883042

3 (months-old* or 1-y*-old* or one-y*-old* or 2-y*-old* or two-y*-old* or 3-y*-old* or three-y*-old* or 4-y*-old* or four-y*-old* or 5-y*-old* or five-y*-old* or 6-y*-old* or six-y*-old* or 7-y*-old* or seven-y*-old* or 8-y*-old* or eight-y*-old* or 9-y*-old* or nine-y*-old* or 10-y*-old* or ten-y*-old* or 11-y*-old* or eleven-y*-old* or 12-y*-old* or twelve-y*-old* or 13-y*-old* or thirteen-y*-old* or 14-y*-old* or fourteen-y*-old* or 15-y*-old* or fifteen-y*-old* or 16-y*-old* or sixteen-y*-old* or 17-y*-old* or seventeen-y*-old*).mp. 296848

4 2 or 3 5929180

5 1 and 4 2036

6 ("chronic non-bacterial osteomyelitis" or "chronic nonbacterial osteomyelitis" or "chronic recurrent multifocal osteomyelitis" or "chronic recurrent multi-focal osteomyelitis" or mandib* or jaw or jaws or sclerosing).ti,bt,kf. 82032

7 5 not 6 1384

8 Osteomyelitis.ti,bt,kf. 17998

9 Osteomyelitis/ 22806

10 8 or 9 26693

11 7 and 10 1078

12 (case reports/ or (case-stud* or case-report*).jw. or (case-study or (case-report not case-report form*)).mp.) not (registry or registries or "case reports" or "case studies" or "case series" or "case control" or "concurrent cases" or consecutive case* or ((multiple or several or "2*" or "3*" or "4*" or "5*" or "6*" or "7*" or "8*" or "9*" or "10*" or "11*" or "12*" or "13*" or "14*" or "15*" or "16*" or "17*" or "18*" or "19*" or four or five or six or seven or eight or nine or ten or eleven or twelve or thirteen or fourteen or fifteen or sixteen or seventeen or eighteen or nineteen or twenty or thirty or forty or fifty or sixty or seventy or eighty or ninety) adj3 ("cases" or "patients" or "individuals" or "children" or "infants" or "adolescents" or "boys" or "girls"))).tw. 2299767

13 11 not 12 830

14 limit 13 to yr="1980 -Current" 694

**Embase <1974 to 2025 February 12>(OVID interface)**

Date searched: Feb 13, 2025

Results: 653

1 ((recurrent or chronic or persistent) adj3 osteomyelitis).tw,kf. 5977

2 chronic osteomyelitis/ 3447

3 Osteomyelitis.ti,bt,kf. 18151

4 1 and (2 or 3) 4757

5 exp Child/ or exp infant/ or adolescent/ or exp pediatrics/ or (pediatric* or paediatric* or child* or newborn* or congenital* or infan* or baby or babies or neonat* or pre-term or preterm* or premature birth* or NICU or preschool* or pre-school* or kindergarten* or kindergarden* or "elementary school*" or "nursery school*" or ("day care*" not adult*) or schoolchild* or toddler* or boy or boys or girl* or "middle school*" or pubescen* or juvenile* or teen* or youth or youths or "high school*" or "junior high" or adolesc* or pre-pubesc* or prepubesc*).mp. 5803395

6 (months-old* or 1-y*-old* or one-y*-old* or 2-y*-old* or two-y*-old* or 3-y*-old* or three-y*-old* or 4-y*-old* or four-y*-old* or 5-y*-old* or five-y*-old* or 6-y*-old* or six-y*-old* or 7-y*-old* or seven-y*-old* or 8-y*-old* or eight-y*-old* or 9-y*-old* or nine-y*-old* or 10-y*-old* or ten-y*-old* or 11-y*-old* or eleven-y*-old* or 12-y*-old* or twelve-y*-old* or 13-y*-old* or thirteen-y*-old* or 14-y*-old* or fourteen-y*-old* or 15-y*-old* or fifteen-y*-old* or 16-y*-old* or sixteen-y*-old* or 17-y*-old* or seventeen-y*-old*).mp. 413817

7 5 or 6 5872108

8 4 and 7 2025

9 ("non-bacterial osteomyelitis" or "nonbacterial osteomyelitis" or "multifocal osteomyelitis" or "multi-focal osteomyelitis" or mandib* or jaw or jaws or sclerosing).ti,bt,kf. 79740

10 8 not 9 1018

11 ((Case report/ and ((We-report or we-describe or we-present or ((year* old or month* old or day* old or yr* old or y old) adj3 (female or male or child or adolescent or girl or boy or baby or infant or patient or individual)) or ((present or describe or report) adj5 case)).ti,ab,kf. or case.ti,bt. or letter.pt.)) or (case-stud* or case-report*).jx. or (case-study or (case-report not case-report-form*)).ti,ab,kf.) not (case-series or case-control or concurrent cases or (("2*" or "3*" or "4*" or "5*" or "6*" or "7*" or "8*" or "9*" or "10*" or "11*" or "12*" or "13*" or "14*" or "15*" or "16*" or "17*" or "18*" or "19*" or four or five or six or seven or eight or nine or ten or eleven or twelve or thirteen or fourteen or fifteen or sixteen or seventeen or eighteen or nineteen or twenty or thirty or forty or fifty or sixty or seventy or eighty or ninety) adj3 (cases or reports or patients or individuals or children or infants or adolescents or boys or girls or adults or older adults or men or women))).mp. 1680011

12 10 not 11 808

13 limit 12 to yr="1980 -Current" 755

14 limit 13 to conference abstracts 102

15 13 not 14 653

**CINAHL Plus with Full Text (EBSCOhost interface)**

Date searched: Feb 13, 2025

Results: 137

S1 ( ((recurrent or chronic or persistent) N3 osteomyelitis) ) AND ( (MH "Osteomyelitis") OR TI(Osteomyelitis) )

S2 ( (MH "Child+") OR (MH "Minors (Legal)") OR (MH "Adolescence+") ) OR ( pediatric* or paediatric* or child* or newborn* or NICU or congenital* or infan* or baby or babies or neonat* or pre-term or preterm* or "premature birth*" or toddler* or preschool* or pre-school* or "nursery school*" or "day care*" or daycare or kindergarten* or kindergarden* or schoolchild* or "elementary school*" or "primary-school*" or "middle school*" or "junior high" or "senior high" or "high school*" or *boy or boys or girl* or pubescen* or juvenile* or teen* or youth or youths or adolesc* or pre-pubesc* or prepubesc* ) OR ( months-old* or 1-y*-old* or one-y*-old* or 2-y*-old* or two-y*-old* or 3-y*-old* or three-y*-old* or 4-y*-old* or four-y*-old* or 5-y*-old* or five-y*-old* or 6-y*-old* or six-y*-old* or 7-y*-old* or seven-y*-old* or 8-y*-old* or eight-y*-old* or 9-y*-old* or nine-y*-old* or 10-y*-old* or ten-y*-old* or 11-y*-old* or eleven-y*-old* or 12-y*-old* or twelve-y*-old* or 13-y*-old* or thirteen-y*-old* or 14-y*-old* or fourteen-y*-old* or 15-y*-old* or fifteen-y*-old* or 16-y*-old* or sixteen-y*-old* or 17-y*-old* or seventeen-y*-old* )

S3 S1 AND S2

S4 TI("non-bacterial osteomyelitis" or "nonbacterial osteomyelitis" or "multifocal osteomyelitis" or "multi-focal osteomyelitis" or mandib* or jaw or jaws or sclerosing)

S5 S3 NOT S4

S6 ((MH "Case Studies") OR SO(case-stud* or case-report*) OR (case-study or ("case report" not "case-report form*")) ) NOT (registry or registries or "case reports" or "case studies" or "case series" or "case control" or "concurrent cases" or consecutive case* or ((multiple or several or "2*" or "3*" or "4*" or "5*" or "6*" or "7*" or "8*" or "9*" or "10*" or "11*" or "12*" or "13*" or "14*" or "15*" or "16*" or "17*" or "18*" or "19*" or four or five or six or seven or eight or nine or ten or eleven or twelve or thirteen or fourteen or fifteen or sixteen or seventeen or eighteen or nineteen or twenty or thirty or forty or fifty or sixty or seventy or eighty or ninety) N3 ("cases" or "patients" or "individuals" or "children" or "infants" or "adolescents" or "boys" or "girls")))

S7 S5 NOT S6 Limiters - Publication Date: 19800101-

**Scopus (Advanced Search)**

Date searched: Feb 13, 2025

Results: 705

( ( TITLE-ABS ( ( recurrent OR chronic OR persistent ) W/3 osteomyelitis ) AND ( TITLE ( osteomyelitis ) OR KEY ( "chronic osteomyelitis" ) ) ) AND TITLE-ABS-KEY(pediatric* or paediatric* or child* or newborn* or congenital* or infan* or baby or babies or neonat* or pre-term or preterm* or "premature birth*" or NICU or preschool* or pre-school* or kindergarten* or kindergarden* or "elementary school*" or "nursery school*" or ("day care*" AND NOT adult*) or schoolchild* or toddler* or boy or boys or girl* or "middle school*" or pubescen* or juvenile* or teen* or youth or youths or "high school*" or "junior high" or "senior high" or adolesc* or young-adult* or pre-pubesc* or prepubesc* or months-old* or 1-y*-old* or one-y*-old* or 2-y*-old* or two-y*-old* or 3-y*-old* or three-y*-old* or 4-y*-old* or four-y*-old* or 5-y*-old* or five-y*-old* or 6-y*-old* or six-y*-old* or 7-y*-old* or seven-y*-old* or 8-y*-old* or eight-y*-old* or 9-y*-old* or nine-y*-old* or 10-y*-old* or ten-y*-old* or 11-y*-old* or eleven-y*-old* or 12-y*-old* or twelve-y*-old* or 13-y*-old* or thirteen-y*-old* or 14-y*-old* or fourteen-y*-old* or 15-y*-old* or fifteen-y*-old* or 16-y*-old* or sixteen-y*-old* or 17-y*-old* or seventeen-y*-old*) **AND PUBYEAR > 1979 AND PUBYEAR < 2026**) AND NOT (TITLE("non-bacterial osteomyelitis" or "nonbacterial osteomyelitis" or "multifocal osteomyelitis" or "multi-focal osteomyelitis" or mandib* or jaw or jaws or sclerosing) OR (( SRCTITLE ( case-stud* OR case-report* ) OR TITLE-ABS-KEY ( {case study} OR {case report} ) ) AND NOT TITLE-ABS-KEY ( {case-series} OR {case-control} OR "2* case*" OR "3* case*" OR "4* case*" OR "5* case*" OR "6* case*" OR "7* case*" OR "8* case*" OR "9* case*" OR "10* case*" OR "11* cases" OR "12* cases" OR "13* cases" OR "14* cases" OR "15* cases" OR "16* cases" OR "17* cases" OR "18* cases" OR "19* cases" OR ((four or five or six or seven or eight or nine or ten or eleven or twelve or thirteen or fourteen or fifteen or sixteen or seventeen or eighteen or nineteen or twenty OR thirty or forty or fifty OR sixty OR seventy OR eighty OR ninety) W/2 cas
